# Optimal combination of immune cells for predicting outcomes in ESCC patients following neoadjuvant immunochemotherapy: Results from a prospective phase II trial

**DOI:** 10.1016/j.gendis.2026.102040

**Published:** 2026-01-16

**Authors:** Peipei Wang, Yueyun Chen, Lin Lu, Yue Zheng, Xia Liu, Haibo Mao, Zexin Yi, Jiajun Li, Qin Zhang, Chengwu Zeng, Yong Wu, Zhenyu Ding, Cunte Chen

**Affiliations:** a Department of Oncology, School of Medicine, the Second Affiliated Hospital of South China University of Technology (Guangzhou First People's Hospital), Guangzhou, Guangdong 510180, China; b Department of Biotherapy, Cancer Center, West China Hospital, West China Medical School, State Key Laboratory of Biotherapy, Sichuan University, Chengdu, Sichuan 610041, China; c Department of Postgraduate Students, West China Hospital, Sichuan University, Chengdu, Sichuan 610041, China; d Department of Hematology, The Fifth Affiliated Hospital, Guangzhou Medical University, Guangzhou, Guangdong 510700, China; e Department of Hematology, Guangzhou First People's Hospital, Institute of Blood Transfusion and Hematology, Guangzhou Medical University, Guangzhou, Guangdong 510180, China

Neoadjuvant immune checkpoint inhibitor plus chemotherapy (NeoICT) has achieved great success in patients with esophageal squamous cell carcinoma (ESCC).^1^^,^^2^ However, the results of the phase II clinical trial (ChiCTR2100045722) we previously published suggest that the pathological complete response rate for locally advanced ESCC patients receiving neoadjuvant camrelizumab plus chemotherapy is 27.0%.^3^ The clinical outcomes remain heterogeneous, potentially attributed to the local tumor immune microenvironment and anti-tumor T cell responses.^3^, ^4^, ^5^ Our trial suggests better tumor regression staging (TRS) than existing clinical grading methods in evaluating the clinical outcomes of ESCC patients receiving NeoICT and guiding further choice of treatment.^3^ The aim of this study was to explore the association of immune cell subtypes with TRS and to screen for prognostic immune cell combinations using single-cell RNA sequencing, bulk RNA sequencing, and multiplexed immunofluorescence techniques (Fig. S1). The detailed clinical characteristics are listed in Table S1.

The results indicated that nine distinct cell subsets were annotated with marker genes in ESCC patients receiving NeoICT, including smooth muscle cells, endothelial cells, epithelial cells, myofibroblasts, T cells, macrophage M2, mast cells, B cells, and plasma cells (Fig. 1A; Fig. S2A–S2F). Interestingly, compared with TRS 0/1, the proportion of T cells (29.3% *vs*. 18.6%), epithelial cells (10.9% *vs*. 2.6%), and macrophage M2 (13.8% *vs*. 3.9%) in the TRS 2/3 group significantly increased, but heterogeneity (Fig. 1A; Fig. S2G). Therefore, further analysis is needed for them. UMAP profiles showed significant epithelial cell clustering between TRS 0/1 and 2/3; thus, the TRS 0/1 group was used as a control for inferred large-scale copy number variation analysis to determine abnormal karyotype of epithelial cells in TRS 2/3 (Fig. S3A and S3B). As expected, compared with TRS 0/1, all 22 autosomes in the TRS 2/3 group showed gene copy number amplification or deletion, indicating that malignant epithelial cells in TRS 2/3 have not been completely eliminated after NeoICT (Fig. S3B). So, the immune microenvironment behind it needs further exploration. For macrophage M2, as consistently elevated in the TRS 2/3 group, the macrophage_M2 score significantly increased in the TRS 2/3 group (*p* < 0.001; Fig. S3C). Finally, Kyoto Encyclopedia of Genes and Genomes (KEGG) pathways indicated that compared with TRS 2/3, the macrophage M2-related immune and metastatic pathways in TRS 0/1 were more inhibited (Fig. S3D). For more subpopulations of T cells, further clustering is crucial to explain the differences between TRS 0/1 and 2/3.

**Figure 1:**
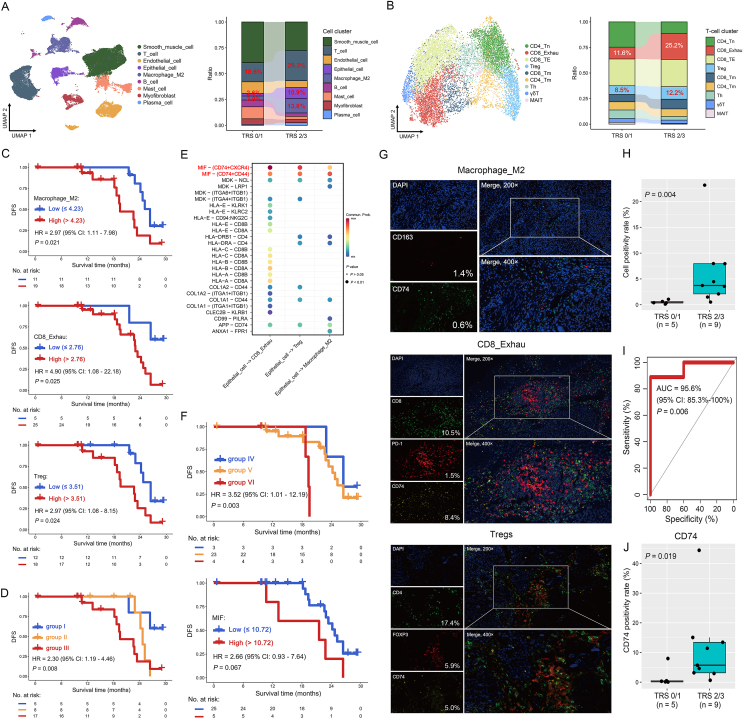
Disease-free survival (DFS) analysis of macrophage_M2, CD8_Exhau, and Tregs. **(A)** UMAP plot of 54,399 cells profiled (left panel) and differences in cell types between TRS 0/1 and 2/3 subgroups (right panel) here, with each cell color-coded for the cell type detected in that cell. **(B)** UMAP plot of 12,299 T cells (left panel) and differences in T-cell types between TRS 0/1 and 2/3 subgroups (right panel). **(C)** Kaplan–Meier survival curves for ESCC patients following NeoICT with low and high infiltration of macrophage_M2 (upper panel), CD8_Exhau (middle panel), and Tregs (bottom panel) cells. **(D)** The effect of combined macrophage-M2, CD8 Exhau, and Tregs on DFS in ESCC patients. Group I: macrophage_M2^low^/CD8_Exhau^low^/treg^low^; Group II: macrophage_M2^high^, CD8_Exhau^high^, or treg^high^; Group III: macrophage_M2^high^/CD8_Exhau^high^/treg^high^. **(E)** Bubble plot showing the ligand–receptor interactions between epithelial cells and macrophage_M2, CD8_Exhau, and Tregs. *p* values are indicated by circle size, and the color scale from blue to red represents the probability of communication from small to large. **(F)** Kaplan–Meier survival curves for ESCC patients with low and high expression levels of macrophage migration inhibitory factor (MIF) signaling pathway (upper panel), as well as the effect of combined CD74, CD44, and CXCR4 on DFS in ESCC patients (bottom panel). Group IV: CD74^low^/CD44^low^/CXCR4^low^; Group V: CD74^high^, CD44^high^, or CXCR4^high^; Group VI: CD74^high^/CD44^high^/CXCR4^high^. **(G)** Representative figures of highly infiltrating macrophage_M2 (upper panel), CD8-Exhau (middle panel), and Tregs (right panel) in the TRS 2/3 group. **(H, I)** The differences in cell positivity rates of macrophage_M2, CD8-Exhau, and Tregs combinations between TRS 0/1 and 2/3 groups (H), and the ROC curve used to predict the performance of TRS 2/3 (I). **(J)** The differences in CD74 expression level between the TRS 0/1 and 2/3 groups.

Nine subpopulations of T cells were identified, and the proportion of CD8-Exhau (25.2% *vs*. 11.6%) and Tregs (12.2% *vs*. 8.5%) in the TRS 2/3 group was higher than that in the TRS 0/1 group, indicating that these two cell subsets play an important role in reducing the response of ESCC patients to NeoICT (Fig. 1B; Fig. S4A–S4C). However, CD8_Exhau and Tregs also exhibited heterogeneity in six patients (Fig. S4D). Particularly, the T cell exhaustion score was significantly increased in the TRS 2/3 group (*p* < 0.001; Fig. S4E). Furthermore, the CD8_Exhau-related immune pathways in TRS 0/1 were more inhibited compared with TRS 2/3 (Fig. S4F). Additionally, the elevated Tregs score was also shown in the TRS 2/3 group (*p* < 0.001; Fig. S4G). Intriguingly, the Tregs-related immune pathways in TRS 2/3 were more activated (Fig. S4H). Altogether, exhausted CD8^+^ T cells and Tregs may contribute to poor response to NeoICT in ESCC patients.

Then, the impact of macrophage_M2, CD8_Exhau, and Tregs on the clinical outcomes of resectable locally advanced ESCC patients treated with NeoICT using 30 bulk RNA-seq data was further explored (Fig. S5A). Critically, the TRS 2/3 group exhibited a higher infiltration of macrophage_M2, CD8_Exhau, and Tregs into the tumor compared with the TRS 0/1 group (*p* < 0.01; Fig. S5B; Table S2). Importantly, high infiltration of macrophage_ M2, CD8-Exhau, and Tregs were significantly associated with poor disease-free survival in ESCC patients receiving NeoICT based on the optimal cut-point (*p* < 0.05; Fig. S5C and S6; Fig. 1C). The combination of multiple variables typically predicts clinical outcomes of patients better than a single one, and the results suggested that the disease-free survival in patient with macrophage_M2^high^/CD8_Exhau^high^/Tregs^high^ was worst (*p* = 0.008; Fig. 1D). Accordingly, macrophage_M2, CD8-Exhau, and Tregs may be potential immune biomarkers for predicting poor prognosis in ESCC patients receiving NeoICT.

Complex cell–cell interactions were observed between epithelial cells and macrophage_M2, CD8_Exhau, and Tregs. Of note, macrophage_M2, CD8_Exhau, and Tregs exhibited relatively high expression of immune response genes (including CD74, CD44, and CXCR4), while corresponding genes, especially macrophage migration inhibitory factor (MIF), were significantly up-regulated in epithelial cells (*p* < 0.01; Fig. 1E). CD8_Exhau and Tregs might be mediated through the MIF-CD74+CXCR4 signaling axes, while macrophage_M2 might be mediated through the MIF-CD74 + CD44 signaling axe (Fig. 1E). Furthermore, in the MIF signaling pathway, there was also strong crosstalk between macrophage_M2, CD8-Exhau, and Tregs in ESCC patients (Fig. S7A and S7B). We inferred that these immune response communications played a crucial role in promoting macrophage_M2, CD8-Exhau, and Tregs infiltration of ESCC in response to NeoICT. Next, the prognostic significance of the MIF pathway profile for ESCC patients receiving NeoICT was investigated using 30 bulk RNA-seq datasets. There was a clear trend indicating that high expression of CD74 was associated with poor disease-free survival in ESCC patients receiving NeoICT, although it was not statistically significant at that point (*p* = 0.081; Fig. S7C). However, the expression levels of CXCR4 and CD44 were not associated with disease-free survival in ESCC patients (*p* > 0.1; Fig. S7C). Interestingly, co-expression of CD74, CD44, and CXCR4 on macrophage_M2, CD8 Exhau, and Tregs significantly predicted poor disease-free survival in ESCC patients receiving NeoICT (*p* = 0.003; Fig. 1F). Moreover, a clear trend suggested that increased expression of MIF correlated with poor disease-free survival in ESCC patients, although it was not statistically significant at that point (*p* = 0.067; Fig. 1F). In summary, the MIF signaling pathway increased crosstalk between macrophage M2, CD8-Exhau, Tregs, and epithelial cells, and predicted clinical outcomes in ESCC patients receiving NeoICT.

Finally, the differences in infiltration levels between TRS 0/1 and 2/3 groups were further validated through multiplexed immunofluorescence techniques. No significant difference was found in macrophage_M2 between TRS 0/1 and 2/3 groups (*p* = 0.240), while CD8-Exhau (*p* = 0.042) and Tregs (*p* = 0.083) showed increased infiltration in TRS 2/3 group (Fig. 1G; Fig. S8). Importantly, when the above three cell subtypes were combined, they significantly increased in the TRS 2/3 group (*p* = 0.004) and exhibited good predictive performance (AUC = 95.6%; 95% CI: 85.3%–100%; *p* = 0.006; Fig. 1H and I). As CD74 was expressed in macrophage_M2, CD8-Exhau, and Tregs and was a receptor for MIF, the expression level of CD74 was further investigated. Interestingly, compared with the TRS 0/1 group, CD74 expression was higher in the TRS 2/3 group (*p* = 0.019; Fig. 1J). Accordingly, the combination of macrophage_M2, CD8-Exhau, and Tregs was better at predicting poor response to NeoICT in ESCC patients than a single one. Remarkably, the increased interaction between tumor cells and macrophage_M2, CD8_Exhau, and Tregs might be mediated through the MIF/CD74 signaling pathway, which established an immunosuppressive microenvironment to promote tumor growth and resistance to ICI in ESCC.

In conclusion, the increased intra-tumor infiltration of macrophages M2, exhausted CD8^+^ T cells, and Tregs identified a subgroup of patients with ESCC who derived less benefit from NeoICT, which presented novel insights for future studies to examine its clinical validity, thereby guiding intensive treatment and clinical trials, and their transition from bench to bedside.

## CRediT authorship contribution statement

**Peipei Wang:** Writing – original draft, Validation, Software, Resources, Investigation, Funding acquisition, Data curation. **Yueyun Chen:** Visualization, Validation, Software, Resources, Methodology, Data curation. **Lin Lu:** Methodology, Formal analysis, Data curation. **Yue Zheng:** Methodology, Formal analysis, Data curation. **Xia Liu:** Methodology, Formal analysis, Data curation. **Haibo Mao:** Methodology, Formal analysis, Data curation. **Zexin Yi:** Methodology, Formal analysis, Data curation. **Jiajun Li:** Methodology, Formal analysis, Data curation. **Qin Zhang:** Resources, Funding acquisition, Formal analysis, Data curation. **Chengwu Zeng:** Writing – review & editing, Supervision, Project administration, Conceptualization. **Yong Wu:** Writing – review & editing, Supervision, Project administration, Methodology, Conceptualization. **Zhenyu Ding:** Writing – review & editing, Supervision, Resources, Funding acquisition, Conceptualization. **Cunte Chen:** Writing – review & editing, Resources, Project administration, Funding acquisition, Conceptualization.

## Ethics declaration

This study was approved by the Ethics Committee of West China Hospital (No. 2020-1179). All participants provided written informed consent.

## Data availability

The datasets used and/or analyzed during the current study are available from the corresponding author on reasonable request.

## Funding

This work was supported by the 10.13039/501100001809National Natural Science Foundation of China (No. 82400223, 82300176), the 10.13039/501100021171Guangdong Basic and Applied Basic Research Foundation (China) (No. 2023A1515110555, 2023A1515012968), Guangzhou Planned Project of Science and Technology (China) (No. 2024A04J3859), the Key R&D Support Plan of Chengdu Science and Technology Bureau in 2023 (China) (No. 2023-YF09-00039-SN), Sichuan Science and Technology Program (China) (No. 2023YFS0001), and the Fundamental Research Funds for the Central Universities (China) (No. 2024ZYGXZR029).

## Conflict of interests

The authors declared no competing interests.
