# Supplementary material for: Optimal combination of immune cells for predicting outcomes in ESCC patients following neoadjuvant immunochemotherapy: Results from a prospective phase II trial
Source: Genes Dis. 2026 Jan 16;13(6):102040. doi: 10.1016/j.gendis.2026.102040 (PMC13355625; doi:10.1016/j.gendis.2026.102040)
Supplement: Multimedia component 1 [file mmc1.docx]

**Optimal combination of immune cells for predicting outcomes in ESCC patients following neoadjuvant immunochemotherapy: results from a prospective phase 2 trial**

**Material and Methods**

**ESCC patients**

In our trial ChiCTR2100045722,^1^ tumor tissues were prospectively collected from 30 resectable locally advanced ESCC patients treated with NeoICT (camrelizumab combined with chemotherapy) for bulk RNA sequencing. A total of 9 patients were assessed as TRS 0/1, while 21 patients were assessed as TRS 2/3. Moreover, tumor tissues were collected from 6 locally advanced ESCC patients treated with NeoICT for single-cell RNA (scRNA) sequencing, including 3 patients assessed as TRS 0/1 and 3 patients assessed as TRS 2/3. In addition, formalin-fixed, paraffin-embedded (FFPE) blocks were collected from 14 locally advanced ESCC patients treated with NeoICT for mIF as validation, including 5 patients assessed as TRS 0/1 and 9 patients assessed as TRS 2/3. These are another group of patients besides scRNA-seq and bulk RNA-seq. Pathologic response after NeoICT was evaluated on surgical samples. Two specialized pathologists followed the Becker criteria for TRS 2. TRS 0 indicates complete (0% residual primary tumor, not including lymph nodes); TRS 1 indicates subtotal tumor regression (<10% residual tumor per tumor bed); TRS 2 indicates partial tumor regression (10-50% residual tumor per tumor bed); TRS 3 indicates minimal or no tumor regression (50% residual tumor per tumor bed). Disease-free survival (DFS) refers to the duration from the date of surgery until the occurrence of any of the following events: disease progression, relapse, or death from any cause. The detailed clinical characteristics are listed in Table S1. This study was approved by the Ethics Committee of West China Hospital (No. 2020-1179). All participants provided written informed consent.

**scRNA sequencing**

Tumor tissues from 6 resectable locally advanced ESCC patients treated with NeoICT were dissociated using the Tumor Dissociation Kit (Miltenyi Biotec, Bergisch Gladbach, Germany) for preparation of single-cell suspension. For scRNA-seq, the cell suspension was loaded into Chromium microfluidic chips with 5' chemistry and barcoded with a 10x Chromium Controller (10X Genomics). RNA from the barcoded cells was subsequently reverse-transcribed and sequencing libraries were constructed with reagents from a Chromium Single Cell 5' reagent kit (10X Genomics) according to the manufacturer's instructions. scRNA sequencing were performed with Illumina (NovaSeq PE150) according to the manufacturer's instructions. For quality control, fastp was used to perform basic statistics on the quality of the raw reads in scRNA-seq. Raw reads were mapped to the human reference genome (hg38), and gene expression was quantified using the “count” subcommands of the Cell Ranger pipeline (10X Genomics) using default parameters.

**Data analysis of scRNA-seq**

Cells with less than 500 genes or more than 4000 genes, as well as mitochondrial gene levels exceeding 15%, were excluded. Biological and technical batch effects of 6 scRNA-seq data were eliminated using the package “Harmony”. The data were integrated using the Seurat 5 integration method, which was used for principal component analysis (PCA) dimensionality reduction analysis. Then, the first 30 dimensions were selected for uniform manifold approximation and projection (UMAP) analysis of the cell clusters by the “FindClusters” and “RunUMAP” functions in Seurat. T-cell subsets were further subgrouped using the above method, and the differentially expressed genes were identified using the Wilcox method in the “FindAllMarkers” function. Specifically, smooth muscle cells highly expressed genes such as LAMA2, PDGFRA, IGF1, and SCARA5. Endothelial cells expressed genes including PECAM1, VWF, AQP1, PLVAP, PTPRB, FLT1, LYVE1, PROX1, CD36, and STAB2, while epithelial cells exhibited elevated expression of KRT6A, KRT16, KRT6B, KRT17, KRT14, KRT5, and KRT19. Myofibroblast was marked by ACTA2, MUSTN1, TAGLN, MYH11, and MYL9. T cells displayed higher levels of CD3D, CD3E, CD3G, and CD247. Macrophage M2 was marked by high expression of CD163, CD68, VSIG4, F13A1, CCL18, MRC1, and MSR1. Mast cells showed higher expression of TPSD1, TPSAB1, TPSB2, CPA3, and KIT. B cells exhibited elevated expression of CD19, CD79A, PAX5, CD79B, and CD22, while plasma cells displayed higher levels of IGHG1, IGKC, MZB1, TNFRSF17, FCRL5, POU2AF1, CD38, and XBP1. Moreover, the marker genes for naïve CD4+ T cells (CD4_Tn) were CD4, LEF1, TCF7, SELL, and CCR7. Exhausted CD8+ T cells (CD8_Exhau) high expression of inhibitory molecules (HAVCR2, LAG3, CTLA4, PDCD1, TOX, CXCL13, LAYN, and CXCR6). Terminal effect CD8+ T cells (CD8_TE) highly expressed cytotoxic markers (GZMK, GZMM, GZMH, GZMA, IFNG, and NKG7), while low in memory CD8+ T cells (CD8_Tm) and memory CD4+ T cells (CD4_Tm). Tregs were characterized by FOXP3, IL2RA, IKZF2, and CCR8, and T helper cells (Th) were identified by IL7R, CD40LG, and KLRB1. γδT cells exhibited elevated expression of TRDC, KLRC2, KLRC1, KLRF1, and KLRD1. Mucosal-associated invariant T cells (MAIT) displayed higher levels of RORC, SLC4A10, IL4I1, IL23R, and LST1.

**Inferring copy number variation (InferCNV) analysis**

The count matrix of epithelial cell clusters derived from the Seurat object was utilized for CNV analysis. The initial CNV value for a single cell in each sample was estimated according to the average expression profile across chromosome intervals using the package “infercnv”. Cell clustering was further performed, with 3 ESCC patients assessed as TRS 0/1 as the control group, and the malignant epithelial cell clusters with higher CNV changes were identified in the other 3 ESCC patients assessed as TRS 2/3. The CNV scores were calculated by the average square of the CNV value across the genome to quantitatively evaluate the CNV level of each single cell.

**Cell-cell interaction analysis**

To systematically analyze the interactions between cells, the package "CellChat" was used to reveal the ligand-receptor pairs between CD8-Exhau, Treg, Macrophage_M2, and epithelial cells of ESCC patients assessed as TRS 2/3 in scRNA-seq data. The significant ligand-receptor pairs were identified with *P* < 0.05.

**Bulk RNA-seq and data processing**

Tumor tissues from 30 resectable locally advanced ESCC patients treated with NeoICT were dissociated for the construction of a cDNA sequencing library. The cDNA library was sequenced using Illumina NovaSeq6000 with PE150 sequencing mode. The raw data undergoes the following steps to filter out unqualified reads with low overall quality or containing sequencing primers or low-end quality: remove all connector sequences contained in the reads; remove bases with a mass Q < 20 at the 3 'end; remove reads with length < 25; remove human ribosome RNA reads. High-quality reads were mapped to the human reference genome (GRCh38) using the split mapping algorithm in Hisat2 (version 2.0.4). The expression level of each gene is calculated as transcripts per million reads (TPM). Finally, based on the average expression levels of differential genes, with |log_2_ (foldchange)| > 1 and *P* < 0.001, between cell types determined in scRNA data, the corresponding cell types for each ESCC patient in bulk-RNA data were calculated.

**mIF detection**

mIF was performed on FFPE tumor tissues from 13 resectable locally advanced ESCC patients treated with NeoICT as previously described.^4^ A validated multiple fluorescence staining kit from Servicebio (Wuhan, China) was used following the manufacturer’s protocol. Macrophage_M2 cells were stained as follows: CD163 (Servicebio, GB115709，1:300), CD74 (Servicebio, GB151179，1:300), and DAPI (nuclear counterstain). Moreover, CD8_Exhau cells were stained as follows: CD8 (Servicebio, GB12068，1:3000), PD-1 (Servicebio, GB15338，1:2000), CD74 (Servicebio, GB151179，1:2000), and DAPI (nuclear counterstain), and Tregs were stained as follows: CD4 (Servicebio, GB13588，1:1000), FOXP3 (Servicebio, GB112325, 1:2000), CD74 (Servicebio, GB151179，1:2000), and DAPI (nuclear counterstain). The sample scanning, spectral unmixing, and quantification of signals were performed with the CaseViewer software (Version 2.3) of 3DHISTECH in the panoramic MIDI Pathology Imaging System.

**Statistical analysis**

All statistical analyses were carried out using R software (version 4.3.2, <https://www.r-project.org/>). Two groups of quantitative variables were compared by a Mann-Whitney-Wilcoxon test. The Kaplan-Meier curves among subgroups were compared using the log-rank test from the package "survival".^5, 6^ The package "survminer" was utilized to determine the optimal prognostic cut-point for quantitative variables through the application of maximally selected rank statistics.^7-9^ The area under the curve (AUC) in the receiver operating characteristic curve was determined by the package "pROC". A two-tailed *P* < 0.05 was considered statistically significant.

**Table S1.** Baseline characteristics.

| Variables | Bulk RNA-seq, N (%) | scRNA-seq, N (%) | mIF, N (%) |
| --- | --- | --- | --- |
| Number | 30 | 6 | 14 |
| Groups |  |  |  |
| TRS 0/1 | 9 (30.0) | 3 (50.0) | 5 (35.7) |
| TRS 2/3 | 21 (70.0) | 3 (50.0) | 9 (64.3) |
| Age, years |  |  |  |
| ≤ 60 | 7 (23.3) | 3 (50.0) | 2 (14.3) |
| > 60 | 23 (76.7) | 3 (50.0) | 12 (85.7) |
| Sex |  |  |  |
| Female | 6 (20.0) | 1 (16.7) | 4 (28.6) |
| Male | 24 (80.0) | 5 (83.3) | 10 (71.4) |
| Smoking history |  |  |  |
| No | 10 (33.3) | 5 (83.3) | 8 (57.1) |
| Yes | 20 (66.7) | 1 (16.7) | 6 (42.9) |
| Alcohol history |  |  |  |
| No | 14 (46.7) | 4 (66.7) | 8 (57.1) |
| Yes | 16 (53.3) | 2 (33.3) | 6 (42.9) |
| Location |  |  |  |
| Upper | 1 (3.3) | 1 (16.7) | 3 (21.4) |
| Middle | 16 (53.3) | 2 (33.3) | 7 (50.0) |
| Lower | 13 (43.3) | 3 (50.0) | 4 (28.6) |
| T stage |  |  |  |
| T2 | 2 (6.7) | 1 (16.7) | 0 (0) |
| T3 | 23 (76.7) | 5 (83.3) | 10 (71.4) |
| T4 | 5 (16.7) | 0 (0) | 4 (28.6) |
| N stage |  |  |  |
| N0 | 2 (6.7) | 0 (0) | 4 (28.6) |
| N1 | 13 (43.3) | 4 (66.7) | 5 (35.7) |
| N2 | 13 (43.3) | 2 (33.3) | 5 (35.7) |
| N3 | 2 (6.7) | 0 (0) | 0 (0) |

N: Regional lymph nodes; TRS: Tumor regression grade. TRS 0 indicates complete regression; TRS 1 indicates near-complete regression; TRS 2 indicates partial regression; TRS 3 indicates negligible or no regression. T: Primary tumor.


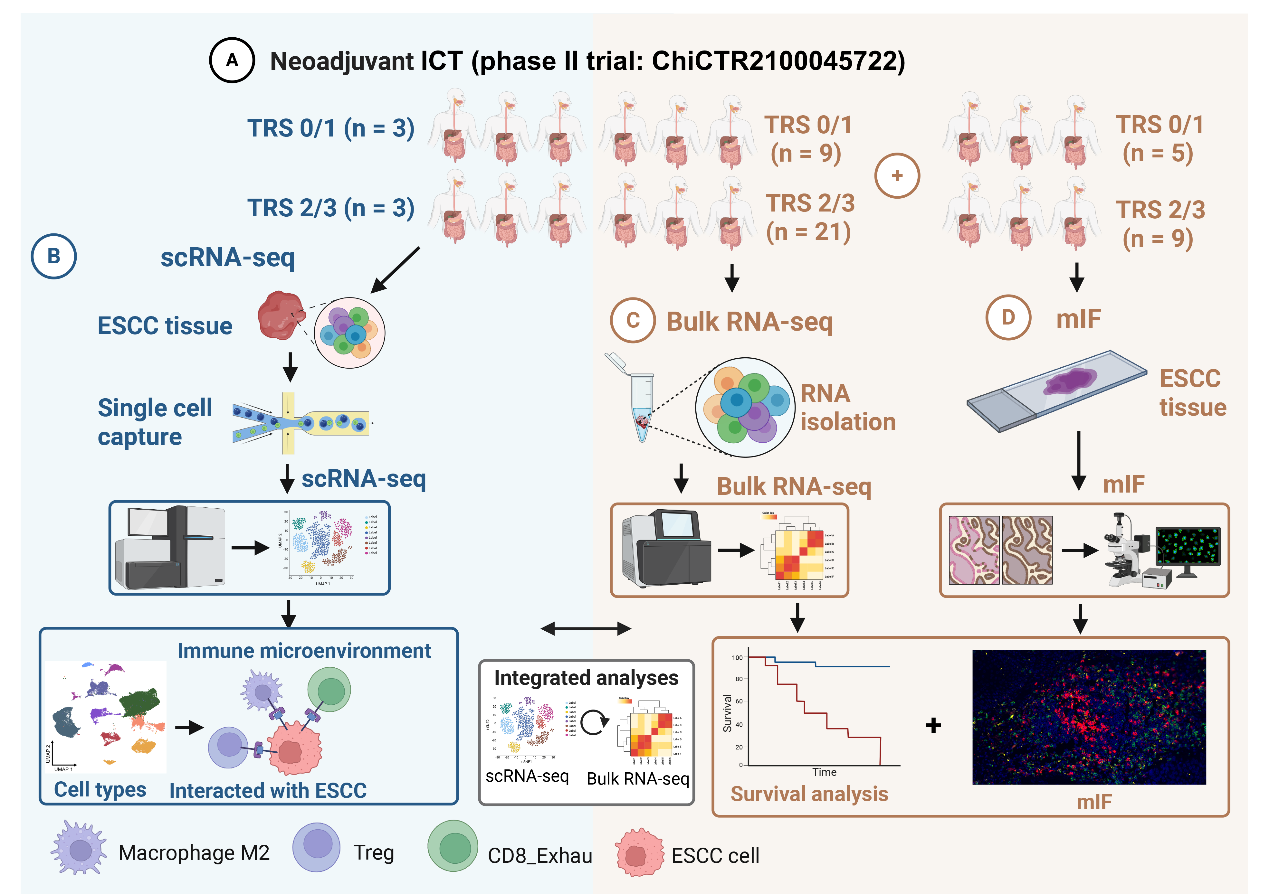


**Figure S1.** Study schematics. Tumor tissues were collected from 6 locally advanced ESCC patients treated with NeoICT for scRNA-seq, 30 patients for bulk RNA-seq, and 13 patients for multiplexed immunofluorescence (mIF) detection. All samples were obtained from a phase II trial (ChiCTR2100045722). Firstly, the differences in immune microenvironment between tumor regression staging (TRS) 2/3 and 0/1 were investigated, followed by integrated analysis with bulk RNA-seq data for prognostic analysis. Finally, the infiltration of immune cells between TRS 0/1 and 2/3 was identified by mIF.


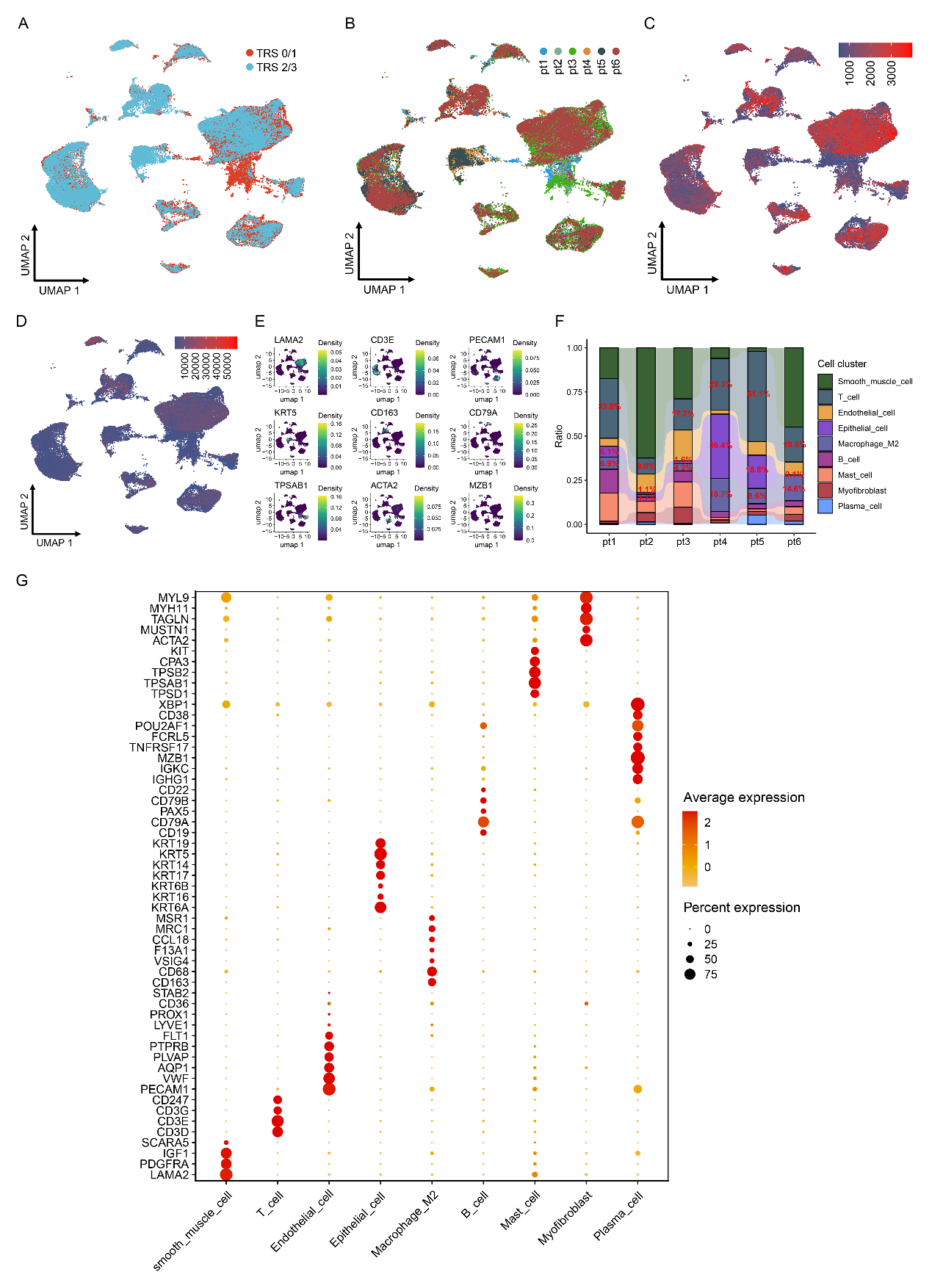


**Figure S2.** Single-cell transcriptional profiling of ESCC patients in the TRS 0/1 and 2/3 subgroups. A-D: UMAP plot of 54,399 cells profiled here, with each cell color-coded for sample type of origin (TRS 0/1 and 2/3) (A), the corresponding patients (B), the number of genes (nFeature) (C) and the number of transcripts (UMIs) (D) detected in that cell. Note that lower-quality cells are discarded from further analyses. (E) Expression of marker genes for the cell types defined above each panel. (F) Bubble plot showing the signature gene expression of the above cell types. (G) Differences in cell types among 6 patients.


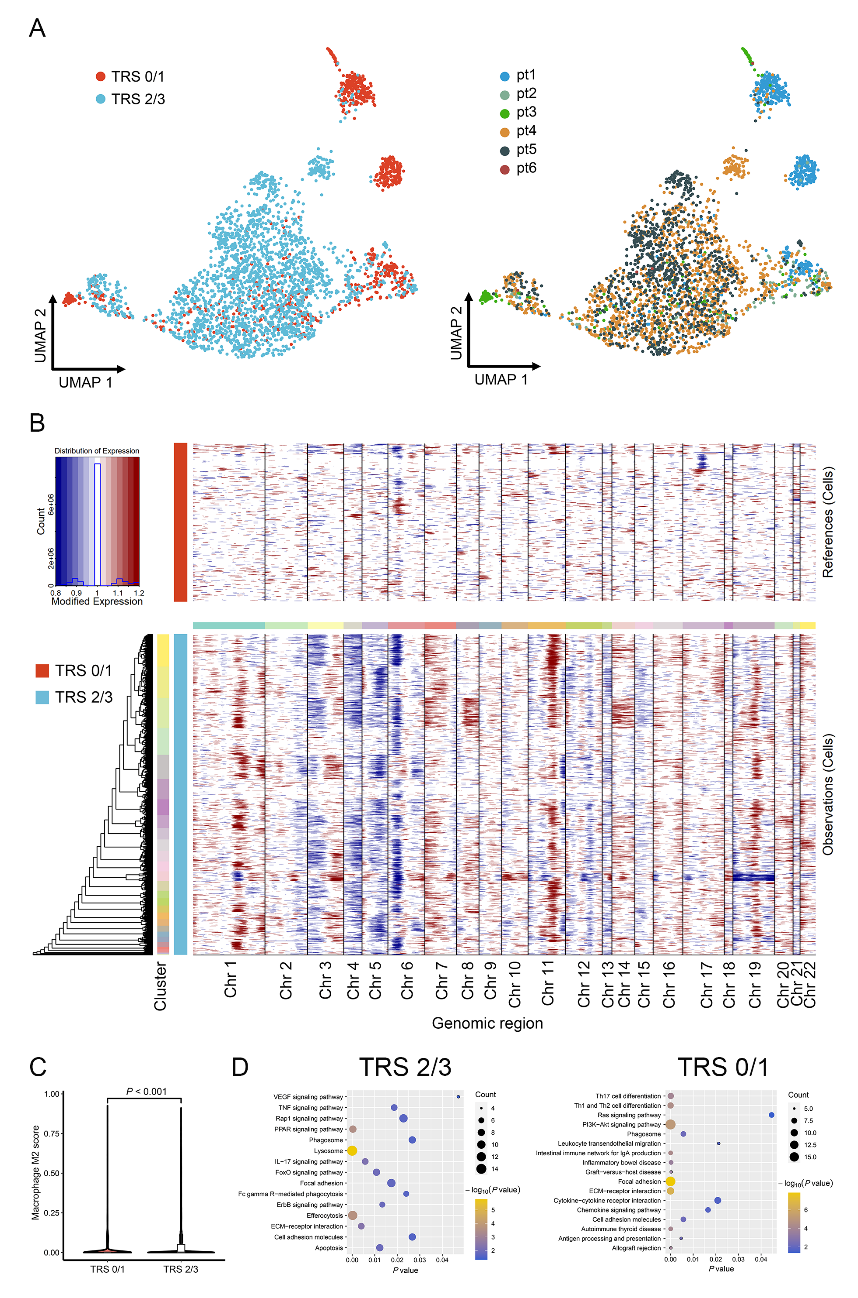


**Figure S3.** The single-cell transcriptomes of epithelial cells and macrophage M2. (A) UMAP plot of 3,116 epithelial cells, color-coded by the sample type of origin (TRS 0/1 and TRS 2/3) (left panel) and the corresponding patients (right panel). (B) Heatmap showing large-scale copy number variations (CNVs) of each malignant epithelial sub-cluster. The expression values for epithelial cells in the TRS 0/1 subgroup are plotted in the upper heatmap, and the cancer cells are plotted in the bottom heatmap, with genes ordered according to the chromosomes. (C) Macrophage M2 score calculated by average expression of marker genes (CD163, CD68, VSIG4, F13A1, CCL18, MRC1, and MSR1) for macrophage M2 were compared between TRS 0/1 and TRS 2/3. Two groups of quantitative variables were compared by a Mann-Whitney-Wilcoxon test. (D) Bubble plots showing the enriched terms by Kyoto Encyclopedia of Genes and Genomes (KEGG) analysis for differentially expressed gene (DEGs) (upregulated pathways shown in TRS 2, downregulated pathways shown in TRS 0) identified by comparing Macrophage_M2 cluster between TRS 2 and TRS 0 with *P* < 0.05. The selection criteria for DEGs are as follows: adjusted *P* < 0.01 and absolute fold change (FC) > 2.


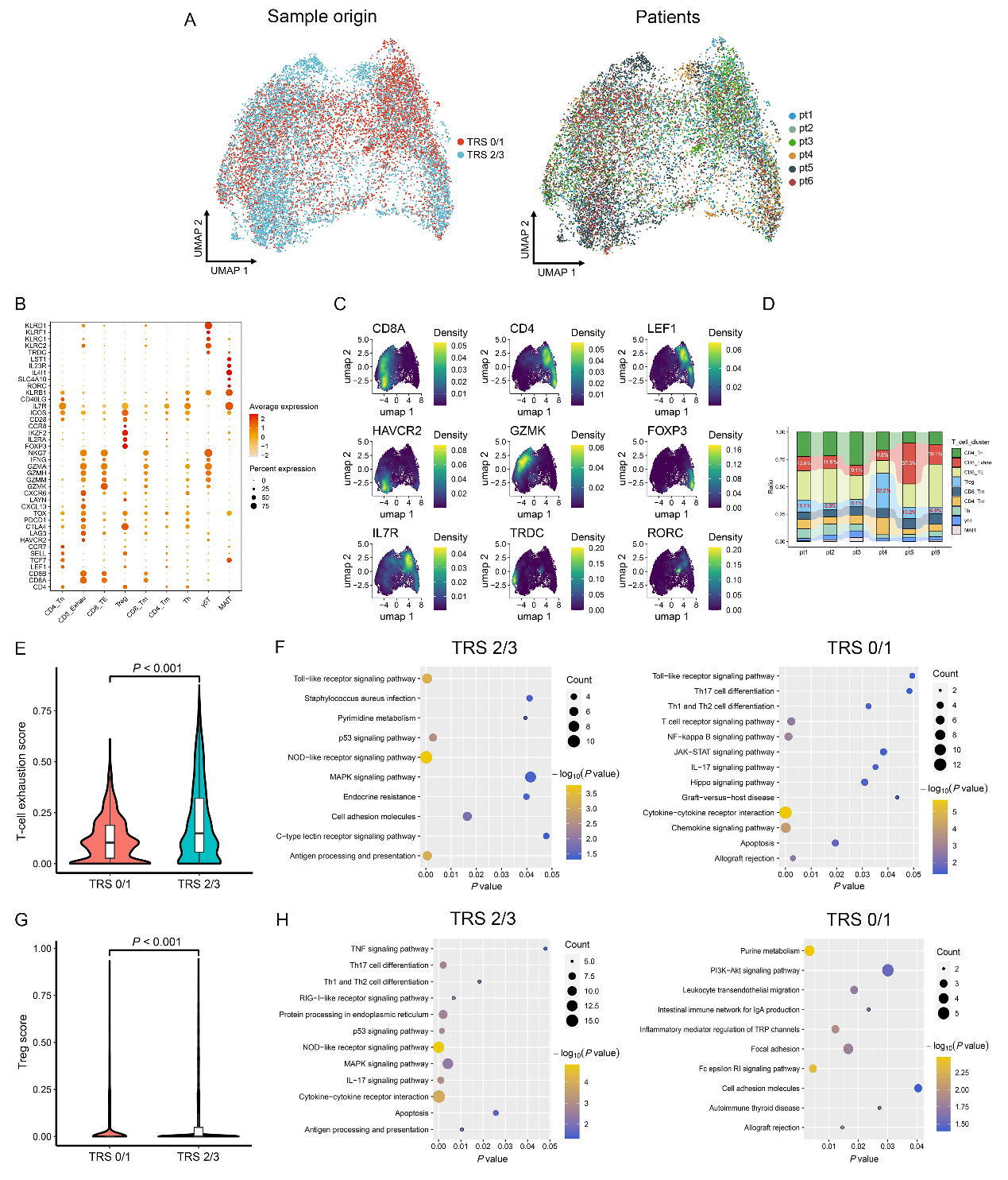


**Figure S4.** Characterization of T cells. (A) UMAP plot of 12,299 T cells, color-coded by the sample type of origin (TRS 0/1 and 2/3) (left panel) and the corresponding patients (right panel). (B) Bubble plot showing the signature gene expression of the T-cell types. (C) Expression of marker genes for the T-cell types defined each panel. (D) Differences in T-cell types among 6 patients. E and G: T-cell exhaustion (E) and Treg (G) scores calculated by average expression of marker genes for CD8_Exhau (HAVCR2, LAG3, CTLA4, PDCD1, TOX, CXCL13, LAYN, and CXCR6) and Treg (FOXP3, IL2RA, IKZF2, and CCR8) were compared between TRS 0/1 and 2/3. Two groups of quantitative variables were compared by a Mann-Whitney-Wilcoxon test. F and H: Bubble plots showing the enriched terms by KEGG analysis for DEGs (upregulated pathways shown in TRS 2/3, downregulated pathways shown in TRS 0/1) identified by comparing matched CD8_Exhau (F) and Treg (H) clusters between TRS 2/3 and 0/1 with *P* < 0.05. The selection criteria for DEGs are as follows: adjusted *P* < 0.01 and absolute FC > 2.


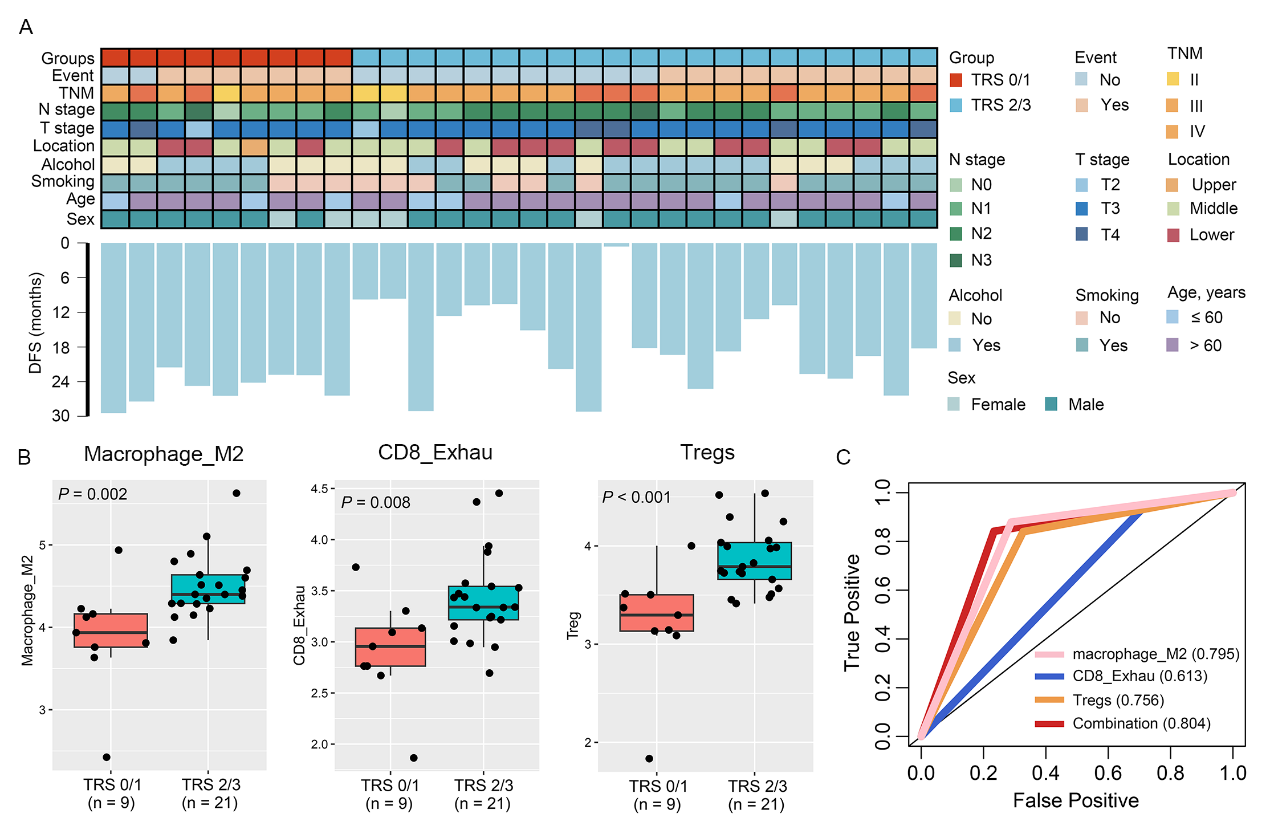


**Figure S5.** Different analysis of macrophage_M2, CD8_Exhau, and Tregs. (A) Baseline clinical characteristics are annotated for each patient. (B) The differences in macrophage_M2 (left panel), CD8_Exhau (middle panel), and Tregs (right panel) between TRS 0/1 and 2/3 subgroups. Macrophage_M2, CD8_Exhau, and Tregs in bulk RNA-seq were estimated by the average expression of cell marker genes in scRNA-seq. Two groups of quantitative variables were compared by a Mann-Whitney-Wilcoxon test. (C) ROC curve was used to evaluate the performance of macrophage_M2, CD8-Exhau, Tregs, and their combinations to predict DFS. ROC curve was plotted using package “survivalROC”.


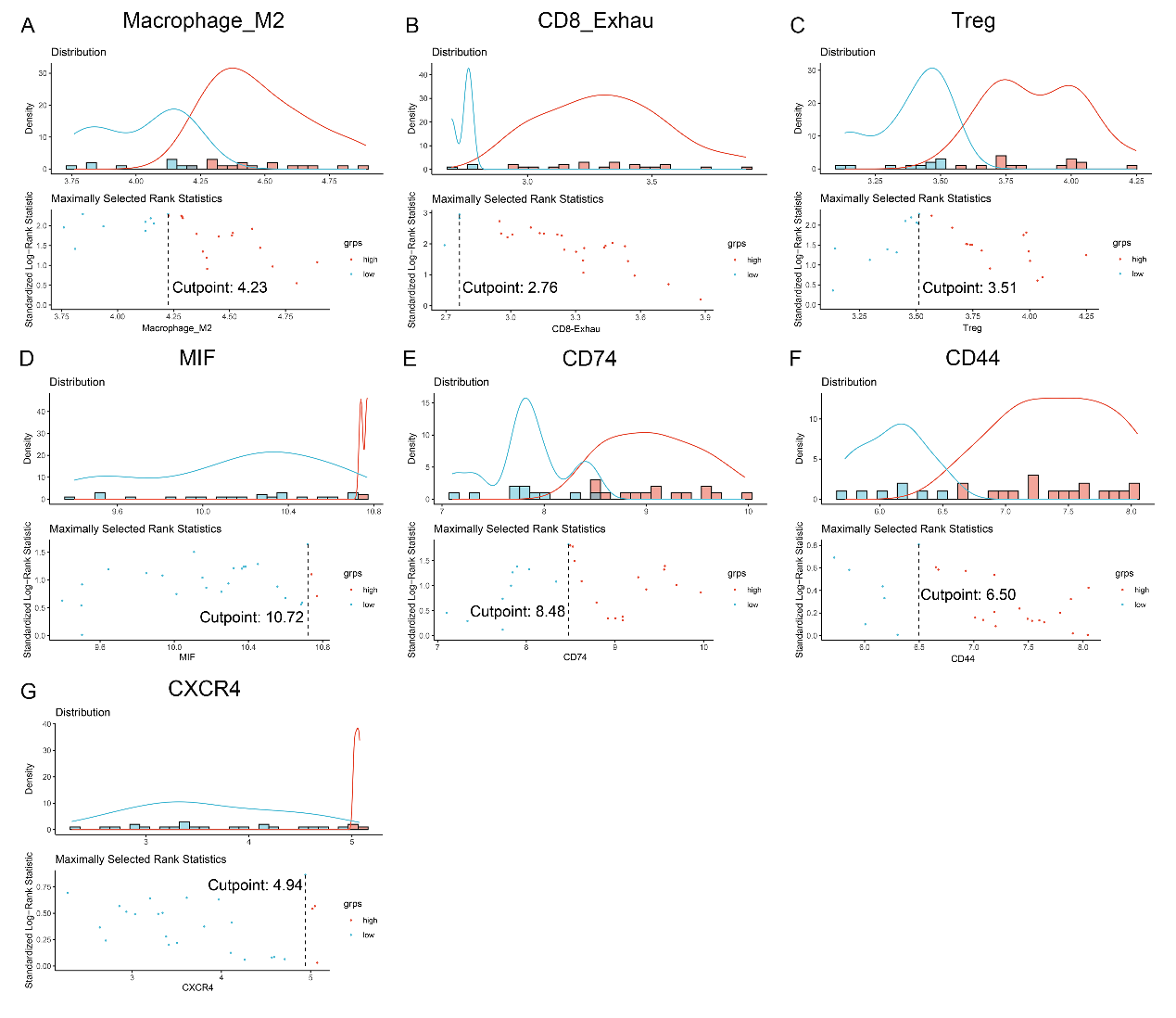


**Figure S6.** The optimal cut-points for macrophage_M2 (A), CD8_Exhau (B), Treg (C), MIF (D), CD74 (E), CD44 (F), and CXCR4 (G).


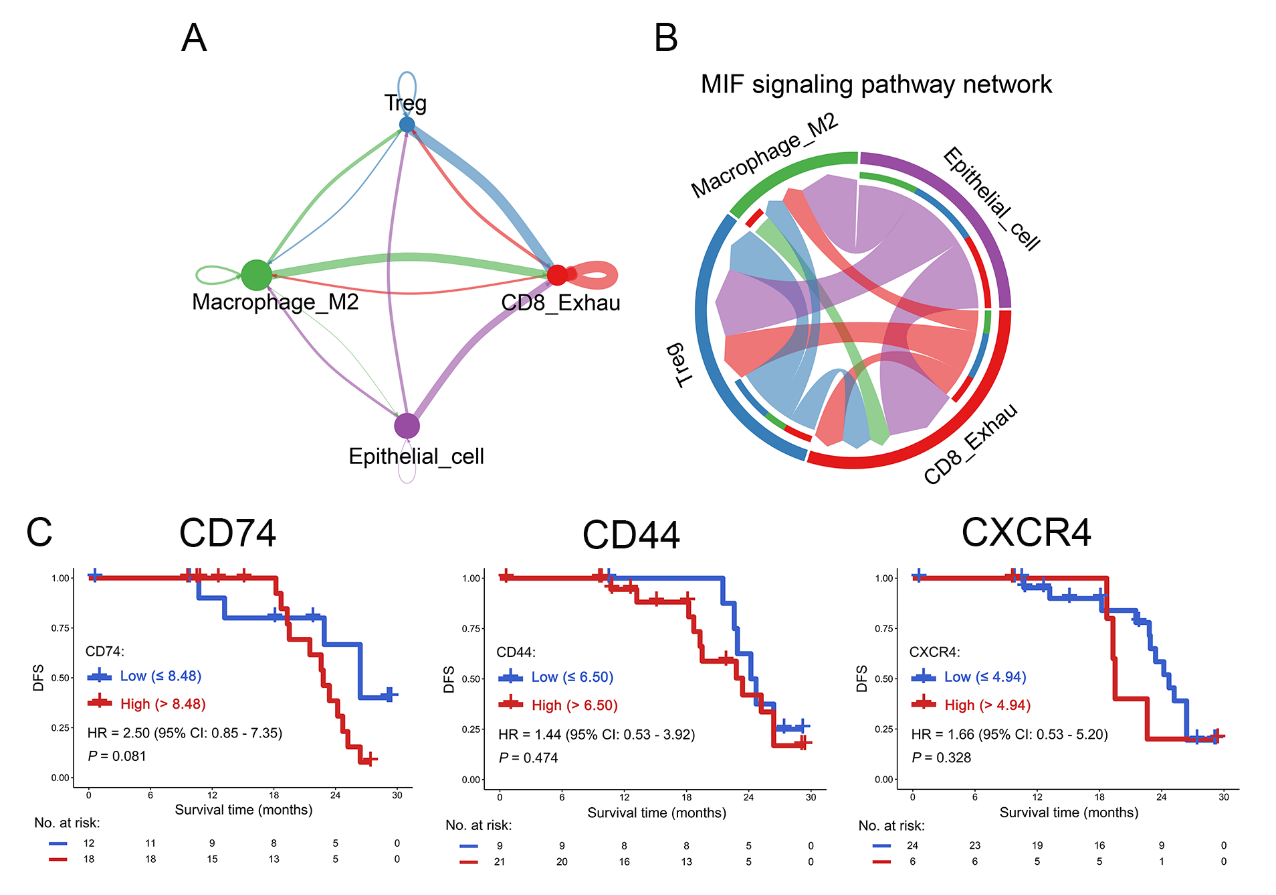


**Figure S7.** Crosstalk between epithelial cells and macrophage_M2, CD8_Exhau and Tregs. A-B: Overview of ligand-receptor interactions of tumor cells and macrophage_M2, CD8_Exhau, and Tregs (A) and Macrophage migration inhibitory factor (MIF) signaling pathway network (B). The line thickness is proportional to the number of ligands when cognate receptors are present in the recipient cell type. The loops indicate autocrine circuits. (C) Kaplan–Meier survival curves for ESCC patients following NeoICT with low and high expression levels of CD74 (left panel), CD44 (middle panel), and CXCR4 (right panel).


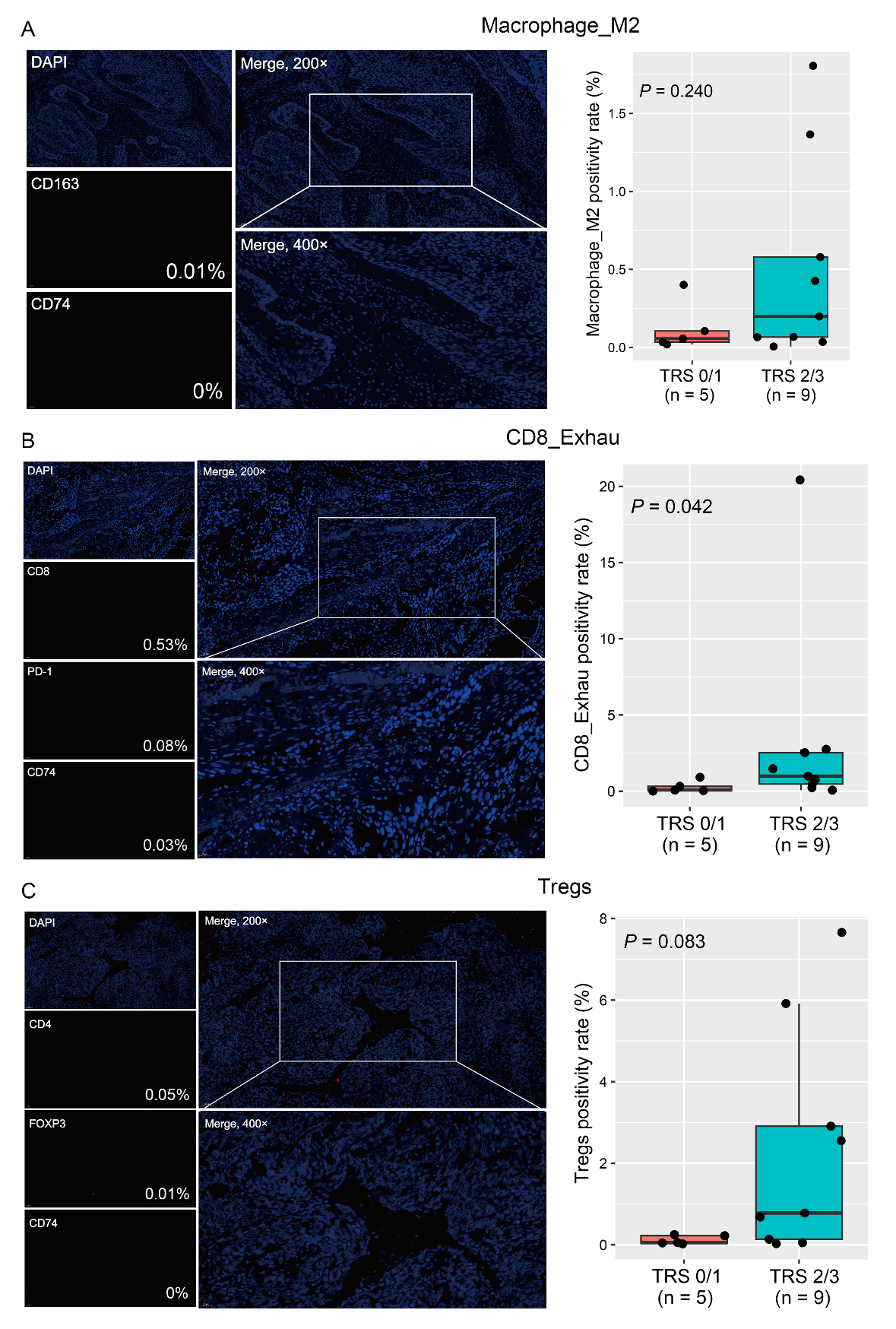


**Figure S8.** Detection of macrophage_M2, CD8_Exhau, Tregs and CD74 expression level by mIF. A-C: Representative figures (left panel) of lowly infiltrating macrophage_M2 (A), CD8-Exhau (B), and Tregs (C) in TRS 0/1 group, and differences in cell positivity rates between TRS 0/1 and 2/3 groups (right panel). Two groups of quantitative variables were compared by a Mann-Whitney-Wilcoxon test.

**Table S2.** The gene lists used to evaluate the average expression level in the bulk RNA-seq

| macrophage_M2 | CD8_Exhau | Tregs |
| --- | --- | --- |
| IL1B | KRT86 | CCR8 |
| CSF3R | ZNF683 | LINC02099 |
| CD163 | LINC02195 | FOXP3 |
| VSIG4 | KIR2DL4 | IL1R2 |
| FPR1 | KLRC2 | TNFRSF4 |
| SERPINA1 | HAVCR2 | RTKN2 |
| CLEC4E | ATP8B4 | IL2RA |
| C1QB | LINC01871 | TBC1D8 |
| FMN1 | CXCL13 | LAIR2 |
| SLC11A1 | DAPK2 | MAGEH1 |
| SMIM25 | LINC02446 | F5 |
| FOLR2 | GZMB | VDR |
| C1QA | CARS | IL12RB2 |
| C1QC | SNAP47 | AC093865.1 |
| SLC16A10 | ITGAE | TNFRSF18 |
| F13A1 | CLIC3 | IKZF2 |
| MS4A7 | SPRY1 | CRADD |
| TLR2 | ITGA1 | GK |
| MS4A4A | ENTPD1 | IL1R1 |
| MS4A6A | PLPP1 | ICA1 |
| LYZ | LAYN | PCBP3 |
| CXCL8 | SRGAP3 | BATF |
| FPR3 | AC243829.4 | LINC01943 |
| LILRB4 | CXCR6 | TBC1D4 |
| AIF1 | KLRC4 | STAM |
| C5AR1 | PARD6G | FAAH21 |
| CCL18 | LAG3 | DNPH1 |
| IGSF6 | NFIL3 | PMAIP1 |
| LGALS2 | GALNT2 | INPP5F |
| CSF2RA | CD8A | SGPP2 |
| RBM47 | POLR1E | PELI1 |
| HCK | SLA2 | ZC3H12D |
| CSF1R | NELL2 | TRAF3 |
| LILRB2 | PRF1 | PLCL1 |
| FCGR2A | RBPJ | HPGD |
| SLAMF8 | GFOD1 | CTLA4 |
| CLEC7A | SSH1 | THADA |
| NCF2 | ENTPD1-AS1 | CARD16 |
| MRC1 | JAML | LY75 |
| LST1 | CTSW | LTB1 |
| APOC1 | SYTL2 | LAYN1 |
| MMP9 | ALOX5AP | ATP13A3 |
| NLRP3 | CD8B | FBLN7 |
| CD14 | ZEB2 | SLAMF1 |
| IFI30 | RIN3 | LINC02694 |
| MNDA | PDCD1 | GLRX |
| G0S2 | DUSP4 | MAST4 |
| PILRA | KLRD1 | TIAM11 |
| ITGAX | KLRK1 | CREB3L2 |
| SLC1A3 | CD7 | AL136456.1 |
| MPEG1 | GZMA | HS3ST3B1 |
| C15orf48 | CAPG | HTATIP2 |
| TYROBP | STMN1 | CCR61 |
| SPI1 | METRNL | MCF2L2 |
| STAB1 | LINC00299 | 3-Mar |
| CD86 | GABARAPL1 | TNFRSF91 |
| KYNU | ACP5 | BACH1 |
| SPP1 | GCNT1 | GPHN |
| FCER1G | GZMH | GADD45A |
| ACSL1 | CEBPB | DUSP16 |
| PLEK | CTSD | NCF4 |
| CD68 | MXRA7 | IL21R |
| CALHM6 | BCL2L11 | GEM |
| SLC43A2 | CHST12 | FNDC3B |
| FCGR2B | FUT8 | CD41 |
| FBP1 | CD63 | TIGIT |
| APOE | UBASH3B | SAT1 |
| RNASE6 | SIRPG | CORO1B |
| C3AR1 | GNLY | ZC3H7A |
| CYBB | CSGALNACT1 | MALT1 |
| PLAUR | ITGB7 | HERC5 |
| BCL2A1 | ASXL2 | SYNGR2 |
| RNF144B | PTMS | TOX2 |
| MERTK | GPR171 | KLHL2 |
| FGR | ECI2 | JAZF1 |
| C1orf162 | MVB12B | RHBDD2 |
| CCL3 | RGS1 | PHACTR2 |
| HLA-DQA2 | PTPN22 | FCRL3 |
| CTSS | PDE4D | ACP51 |
| RNASE11 | HLA-DQA1 | TYMP |
| TNFSF13 | SLC7A5 | MIR4435-2HG |
| RAB20 |  | VAV3 |
| SLCO2B1 |  | ZNRF1 |
| SLC7A7 |  | ACOT9 |
| IRAK2 |  | LFNG |
| HMOX1 |  | PIM2 |
| DMXL2 |  | NINJ1 |
| CXCL3 |  | LDLRAD4 |
| CPVL |  | UGP2 |
| FGD2 |  | TNFRSF1B |
| GK |  | RAB11FIP1 |
| CCL3L1 |  | TXNDC11 |
| TBXAS1 |  | TNFRSF25 |
| LGMN |  | DUSP41 |
| PSTPIP2 |  | HIVEP1 |
| LAIR1 |  | MAP3K1 |
| HBEGF |  | CD59 |
| CXCL16 |  | IRS2 |
| HLA-DRA |  | ICOS |
| THEMIS2 |  | SEC14L1 |
| CTSZ |  | SNX9 |
| CTSB |  | GLCCI1 |
| HLA-DMB |  | BIRC3 |
| SLC8A1 |  | ARHGEF12 |
| HLA-DPA11 |  | GBP2 |
| SNX10 |  | ATOX1 |
| HLA-DPB1 |  | CNST |
| MSR1 |  | SELL1 |
| SLC31A2 |  | IFI61 |
| NHSL1 |  | SPATS2L |
| CD83 |  | HPRT1 |
| EMILIN2 |  | TNIK |
| TNS3 |  | SESN31 |
| RGL1 |  | GRINA |
| HLA-DQA1 |  | PHTF2 |
| TYMP |  | RYBP |
| SH2B3 |  | TULP4 |
| SLC15A3 |  | CTSC |
| MXD1 |  | IGFLR11 |
| PHACTR1 |  | EPSTI1 |
| ALCAM |  | AC008105.3 |
| GCA |  | MAP3K5 |
| HLA-DRB11 |  | PKM |
| FAM49A |  | ARID5B |
| LGALS9 |  | CD27 |
| CIITA |  | ENTPD11 |
| RASGEF1B |  | ZEB1 |
| CTSD |  | FAM53B |
| PPIF |  | CEP120 |
| HLA-DQB1 |  | SEC11C |
| SHTN1 |  | PVT1 |
| LIPA |  | CMSS1 |
| LYN |  | AC016831.7 |
| SYK |  | VPS54 |
| GRK3 |  | FAS |
| FTL |  | IL32 |
| HLA-DMA1 |  | SMYD3 |
| TFRC |  | SERPINB9 |
| TGFBI |  | PRDX2 |
| ADA2 |  | GCNT11 |
| TTYH3 |  | PTPRJ |
| FGD41 |  | GBP5 |
| ACP5 |  | PRDM2 |
| STX11 |  | CFAP20 |
| MPP1 |  | CCDC167 |
| RASSF4 |  | CD281 |
| PIK3R51 |  | RDX |
| CAPG |  | LEF11 |
| CD4 |  | MICAL2 |
| SLC40A1 |  | ZFAND5 |
| CD741 |  | AFTPH |
| GAB2 |  | TRIM14 |
| SAT1 |  | TMEM154 |
| C9orf72 |  | PTTG1 |
| ARRB21 |  | BCL2 |
| SIRPA |  | SAMHD1 |
| HLA-DRB51 |  | JMY |
| DAPK1 |  | TNIP1 |
| IL18 |  | BTG3 |
| CHKA |  | TAB2 |
| RHBDF2 |  | MSI2 |
| DOCK41 |  | USP15 |
| MFSD1 |  | NAB1 |
| MTSS1 |  | IL18R1 |
| UNC93B1 |  | CD82 |
| SGK1 |  | MAP4K3 |
| NINJ1 |  | TPP1 |
| RAB31 |  | ZNRF2 |
| SRGAP21 |  | NDUFV2 |
| NAIP |  | PBXIP1 |
| SIPA1L1 |  |  |
| ABCA1 |  |  |
| LCP21 |  |  |
| BCAT1 |  |  |
| PLIN2 |  |  |
| PIK3AP1 |  |  |
| GM2A |  |  |
| MAFB1 |  |  |
| WASHC2A |  |  |
| ATP2B1-AS1 |  |  |
| ATP6V1B2 |  |  |
| PAPSS21 |  |  |
| RIPK2 |  |  |
| CPM |  |  |
| TOM1 |  |  |
| RBPJ1 |  |  |
| S100A9 |  |  |
| SLC16A3 |  |  |
| FRMD4B1 |  |  |
| TNFSF13B |  |  |
| PDGFC |  |  |
| FGL2 |  |  |
| EPB41L31 |  |  |
| GRN |  |  |
| ITGB21 |  |  |
| NCF1 |  |  |
| FCHO21 |  |  |
| BID |  |  |
| MAP2K1 |  |  |
| SLC25A371 |  |  |
| GPR1831 |  |  |
| SRGAP2B |  |  |
| PTPRE1 |  |  |
| S100A8 |  |  |
| TET2 |  |  |
| ATP13A3 |  |  |
| STXBP2 |  |  |
| VAMP81 |  |  |
| CHMP1B |  |  |
| IRF8 |  |  |
| NFKB1 |  |  |
| KLHL6 |  |  |
| IRAK31 |  |  |
| IL6R |  |  |
| AP2A2 |  |  |
| NCF4 |  |  |
| NAMPT |  |  |
| BASP1 |  |  |
| HAVCR2 |  |  |
| DENND5A |  |  |
| FNIP21 |  |  |
| EFHD2 |  |  |
| ASAH1 |  |  |
| RIN3 |  |  |
| TNFAIP21 |  |  |
| MGAT1 |  |  |
| FMNL2 |  |  |
| H2AFY |  |  |
| NCOA4 |  |  |
| LITAF |  |  |
| MANBA |  |  |
| LY961 |  |  |
| ALOX5 |  |  |
| UPP11 |  |  |
| DAB21 |  |  |
| JARID2 |  |  |
| CCDC88A |  |  |
| MAN2B1 |  |  |
| MYO1F |  |  |
| GNA13 |  |  |
| RILPL2 |  |  |
| ZNF267 |  |  |
| ABR |  |  |
| PSAP1 |  |  |
| PRKAG2 |  |  |
| HCLS11 |  |  |
| GRINA |  |  |
| IL13RA1 |  |  |
| GPR137B |  |  |
| AP1B1 |  |  |
| VEGFA |  |  |
| NUDT161 |  |  |
| TBC1D14 |  |  |
| DOCK5 |  |  |
| PDE4B1 |  |  |
| OXSR1 |  |  |
| ITSN1 |  |  |
| AP1S21 |  |  |
| RNASET2 |  |  |
| NRP21 |  |  |
| BRI3 |  |  |
| RHOQ |  |  |
| NFKBID |  |  |
| COTL11 |  |  |
| CD84 |  |  |
| PLEKHO1 |  |  |
| LAPTM51 |  |  |
| FERMT31 |  |  |
| PYCARD |  |  |
| MAML3 |  |  |
| IFNGR2 |  |  |
| SOD21 |  |  |
| ATG7 |  |  |
| TNFRSF1B1 |  |  |
| GRB2 |  |  |
| FAM49B1 |  |  |
| CTSH1 |  |  |
| STARD131 |  |  |
| ARHGAP261 |  |  |
| GAA |  |  |
| GNAQ1 |  |  |
| FTH1 |  |  |
| ATP2B11 |  |  |
| IER32 |  |  |
| RGS2 |  |  |
| PDXK |  |  |
| SELENOP1 |  |  |
| PARVG |  |  |
| PLD31 |  |  |
| PPT1 |  |  |
| PFKFB31 |  |  |
| ATP1B3 |  |  |
| ATP6V0B |  |  |
| JDP2 |  |  |
| MYO5A |  |  |
| KIF1B |  |  |
| GPNMB1 |  |  |
| LACTB |  |  |
| CREG1 |  |  |
| RABGEF1 |  |  |
| RAPGEF11 |  |  |
| RNF1491 |  |  |
| PDE4DIP |  |  |
| ARHGAP18 |  |  |
| MYO9B |  |  |
| BLVRB |  |  |
| RAB7A |  |  |
| SOAT1 |  |  |
| METRNL |  |  |
| ARL8B |  |  |
| ABHD12 |  |  |
| ZMIZ11 |  |  |
| NCKAP1L |  |  |
| PLEKHB2 |  |  |
| PREX11 |  |  |
| CTSC |  |  |
| C20orf194 |  |  |
| ADAMTSL4-AS1 | |  |
| OGFRL1 |  |  |
| NPC21 |  |  |
| RNF130 |  |  |
| PELI1 |  |  |
| CAMK1D |  |  |
| CERS6 |  |  |
| BEST1 |  |  |
| SERPINB91 |  |  |
| QKI |  |  |
| PTPRJ1 |  |  |
| ATP6V1C1 |  |  |
| CSTB |  |  |
| FCGRT1 |  |  |
| TAOK3 |  |  |
| PTK2B1 |  |  |
| CTSL1 |  |  |
| ZEB2 |  |  |
| CXCL21 |  |  |
| KDM6B |  |  |
| CHST111 |  |  |
| HNMT1 |  |  |
| TPP1 |  |  |
| ATF6 |  |  |
| IRS2 |  |  |
| RGS19 |  |  |
| CYBA1 |  |  |
| ETS21 |  |  |
| SAP30 |  |  |
| CNDP2 |  |  |
| IGFLR1 |  |  |
| ACSL3 |  |  |
| DPYD |  |  |
| B4GALT1 |  |  |
| LIMS1 |  |  |
| LRMDA1 |  |  |
| NR4A3 |  |  |
| NEAT1 |  |  |
| FHIT |  |  |
| ARPC5 |  |  |
| ATP6V0D1 |  |  |
| TANK1 |  |  |
| PABPC4 |  |  |
| AP2S1 |  |  |
| USP361 |  |  |
| CEP170 |  |  |
| SPTLC21 |  |  |
| PITPNA |  |  |
| IVNS1ABP1 |  |  |
| ACER3 |  |  |
| LCP11 |  |  |
| TRIB11 |  |  |
| SPAG91 |  |  |
| RHOG1 |  |  |
| GLA |  |  |
| PPP1R15B |  |  |
| KCNAB2 |  |  |
| ATOX1 |  |  |
| DBI |  |  |
| MAP2K3 |  |  |
| ATP6V1F |  |  |
| SRGAP11 |  |  |
| CPEB4 |  |  |
| CD3021 |  |  |
| FCHSD21 |  |  |
| MMP191 |  |  |
| NUMB |  |  |
| RNF13 |  |  |
| MOB3A |  |  |
| SRGN1 |  |  |
| PHC2 |  |  |
| MAN1A1 |  |  |
| SH3BP51 |  |  |
| UBE2D1 |  |  |
| STX4 |  |  |
| PLXND11 |  |  |
| SQOR |  |  |
| KCTD121 |  |  |
| GNA12 |  |  |
| S100A11 |  |  |
| PDE8A |  |  |
| ANKS1A1 |  |  |
| GPCPD11 |  |  |
| ARPC1B |  |  |
| CSGALNACT2 |  |  |
| VASP |  |  |
| PTPN1 |  |  |
| GSTO1 |  |  |
| CMTM6 |  |  |
| RGS10 |  |  |
| LHFPL21 |  |  |
| GNS1 |  |  |
| PTPN18 |  |  |
| GRASP1 |  |  |
| NAGK |  |  |
| PSEN1 |  |  |
| PGD |  |  |
| SKAP2 |  |  |
| ADAM17 |  |  |
| WAS1 |  |  |
| LAP31 |  |  |
| CCL4L21 |  |  |
| CORO1C1 |  |  |
| GLIPR2 |  |  |
| RAP2B |  |  |
| WASHC2C |  |  |
| CIAO2A |  |  |
| ATP6V0C |  |  |
| PRKCE1 |  |  |
| AREG |  |  |
| NOTCH2 |  |  |
| AGFG1 |  |  |
| ARAP1 |  |  |
| NABP1 |  |  |
| SUSD61 |  |  |
| ISG15 |  |  |
| TALDO1 |  |  |
| AKR1B1 |  |  |
| REL1 |  |  |
| IL10RA1 |  |  |
| SESTD11 |  |  |
| PAK1 |  |  |
| IQGAP21 |  |  |
| N4BP11 |  |  |
| AC007384.11 |  |  |
| VSIR |  |  |
| LTA4H |  |  |
| GAS71 |  |  |
| DOCK21 |  |  |
| TUT7 |  |  |
| STX7 |  |  |
| DIP2B |  |  |
| ETF1 |  |  |
| TCIRG11 |  |  |
| CNPY3 |  |  |
| ABL22 |  |  |
| RALA |  |  |
| GPSM31 |  |  |
| BMP2K |  |  |
| ITPR21 |  |  |
| CYFIP11 |  |  |
| ZNF3311 |  |  |
| SAMSN11 |  |  |
| C4orf48 |  |  |
| ODF3B |  |  |
| SNX29 |  |  |
| PLEKHM2 |  |  |
| FNDC3B |  |  |
| ATG3 |  |  |
| RCOR1 |  |  |
| PTPN6 |  |  |
| SYNGR21 |  |  |
| HEXA |  |  |
| YWHAH |  |  |
| TKT |  |  |
| SERPINB1 |  |  |
| MAP3K81 |  |  |
| ARPC3 |  |  |
| MBP1 |  |  |
| APLP2 |  |  |
| PARVB1 |  |  |
| ATP6AP1 |  |  |
| HIF1A |  |  |
| ELMO11 |  |  |
| DSE1 |  |  |
| CHCHD10 |  |  |
| DENND1A |  |  |
| LAMTOR2 |  |  |
| ZFAND3 |  |  |
| IER5 |  |  |
| ELL2 |  |  |
| BLVRA |  |  |
| IGF2R |  |  |
| SMS |  |  |
| STK101 |  |  |
| FP671120.4 |  |  |
| SMAP21 |  |  |
| EVI2B1 |  |  |
| ALDH21 |  |  |
| TMSB101 |  |  |
| AC022217.3 |  |  |
| DOCK111 |  |  |
| VOPP1 |  |  |
| EPSTI11 |  |  |
| ARHGAP41 |  |  |
| SBF2 |  |  |
| DNAJB6 |  |  |

**References**

1. Chen YY, Wang PP, Hu Y, et al. Clinical efficacy and immune response of neoadjuvant camrelizumab plus chemotherapy in resectable locally advanced oesophageal squamous cell carcinoma: a phase 2 trial. *Br J Cancer*. 2024; 131(7): 1126-1136. doi: 10.1038/s41416-024-02805-5.

2. Bodenhofer U, Bonatesta E, Horejš-Kainrath C, et al. msa: an R package for multiple sequence alignment. *Bioinformatics*. 2015; 31(24): 3997-3999. doi: 10.1093/bioinformatics/btv494.

3. Wagih O. ggseqlogo: A 'ggplot2' Extension for Drawing Publication-Ready Sequence Logos. [*https://CRANR-projectorg/package=ggseqlogo*](https://CRANR-projectorg/package=ggseqlogo). 2017.

4. Wang P, Chen Y, Long Q, et al. Increased coexpression of PD-L1 and TIM3/TIGIT is associated with poor overall survival of patients with esophageal squamous cell carcinoma. *J Immunother Cancer*. 2021; 9(10): e002836. doi: 10.1136/jitc-2021-002836.

5. Therneau TM, Lumley T, Elizabeth A, et al. survival: Survival Analysis. [*https://CRANR-projectorg/package=survival*](https://CRANR-projectorg/package=survival). 2021.

6. Zheng X, Jin Y, Zhang C, et al. RNA-binding protein Roq modulates the Drosophila STING antiviral immune response. *Cell Investigation*. 2025; 1(1): 100002. doi: 10.1016/j.clnves.2024.100002.

7. Chen C, Liu S, Jiang X, et al. Tumor mutation burden estimated by a 69-gene-panel is associated with overall survival in patients with diffuse large B-cell lymphoma. *Exp Hematol Oncol*. 2021; 10(1): 20. doi: 10.1186/s40164-021-00215-4.

8. Zeng C, Nie D, Wang X, et al. Combined targeting of GPX4 and BCR-ABL tyrosine kinase selectively compromises BCR-ABL+ leukemia stem cells. *Mol Cancer*. 2024; 23(1): 240. doi: 10.1186/s12943-024-02162-0.

9. Wang P, Cai Q, Peng X, et al. Increased co-expression of CTLA4/LAG3 predicted adverse clinical outcomes in patients with T-cell malignancies. *Cell Investigation*. 2025; 1(1): 100004. doi: 10.1016/j.clnves.2024.100004.
